# Supplementary material for: Thermalization rate of polaritons in strongly-coupled molecular systems
Source: Nanophotonics. 2024 Mar 7;13(14):2635–49. doi: 10.1515/nanoph-2023-0800 (PMC11635947; doi:10.1515/nanoph-2023-0800)
Supplement: Supplementary file 1 — Supplementary Material Details [file j_nanoph-2023-0800_suppl_001.pdf]

Supplementary Information for Thermalization rate of polaritons in strongly-coupled molecular systems

Evgeny A. Tereshchenkov
Dukhov Research Institute of Automatics (VNIIA),
22 Sushchevskaya, Moscow 127055, Russia;
Moscow Institute of Physics and Technology, 9 Institutskiy pereulok,
Dolgoprudny 141700, Moscow region, Russia; and
Institute for Theoretical and Applied Electromagnetics,
13 Izhorskaya, Moscow 125412, Russia;

Ivan V. Panyukov, Vladislav Yu. Shishkov,[*] and Evgeny S. Andrianov
Dukhov Research Institute of Automatics (VNIIA),
22 Sushchevskaya, Moscow 127055, Russia; and
Moscow Institute of Physics and Technology, 9 Institutskiy pereulok,
Dolgoprudny 141700, Moscow region, Russia;

Mikhail Misko
Moscow Institute of Physics and Technology, 9 Institutskiy pereulok,
Dolgoprudny 141700, Moscow region, Russia;

Anton V. Zasedatelev[†]
Vienna Center for Quantum Science and Technology (VCQ),
Faculty of Physics, University of Vienna,
Boltzmanngasse 5, 1090 Vienna, Austria

(Dated: January 29, 2024)

I. EMISSION SPECTRUM OF AN ORGANIC FILM

The stationary emission spectrum, $I_{\rm em}(\omega)$, can be calculated as follows [1, 2]

$$I_{\rm em}(\omega) = C_{\rm em}\sum_{m=1}^{N_{\rm mol}} {\rm Re}\int_0^{+\infty} e^{-i\omega\tau}\langle\hat{\sigma}^{(m)\dagger}(t+\tau)\hat{\sigma}^{(m)}(t)\rangle d\tau \tag{I.1}$$

where $t\to\infty$ and $C_{\rm em}$ is the normalization constant. We set $C_{\rm em}$ such that

$$\int_{-\infty}^{+\infty} I_{\rm em}(\omega)d\omega = 1. \tag{I.2}$$

We assume that the weak incoherent pumping supplies the energy to the dressed excitons and do not affect the dressed molecular vibrations. In this case, we can separate contribution of these two subsystems in the two-time correlator form Eq. (I.1)

$$\langle\hat{\sigma}^{(m)\dagger}(t+\tau)\hat{\sigma}^{(m)}(t)\rangle \approx \langle\hat{S}^{(m)\dagger}(t+\tau)\hat{S}^{(m)}(t)\rangle\prod_{j=1}^{N_{\rm vib}}\langle\hat{D}_j^{(m)\dagger}(t+\tau)\hat{D}_j^{(m)}(t)\rangle, \tag{I.3}$$

The analysis of the dynamics of the excitons at $t\to+\infty$ gives [3]

$$\langle\hat{S}^\dagger(t+\tau)\hat{S}(t)\rangle = pe^{-(\gamma_{\rm diss}/2-i\omega_0)\tau}, \tag{I.4}$$

.

To obtain $\langle\hat{D}_j^{(m)\dagger}(t+\tau)\hat{D}_j^{(m)}(t)\rangle$ at $t\to+\infty$, we consider the correlator

$$\langle\hat{R}_j^{(m)}(t+\tau,\mu_1,\mu_2)\hat{D}_j^{(m)}(t)\rangle,$$

where $\hat{R}_j^{(m)}(t+\tau,\mu_1,\mu_2) = e^{\mu_1\hat{B}_j^{(m)\dagger}(t+\tau)}e^{-\mu_2\hat{B}_j^{(m)}(t+\tau)}$. Using the Lindblad equation (31) and the quantum regression theorem [1, 2], we obtain an equation for this correlator

$$\begin{aligned}&\frac{\partial}{\partial\tau}\langle\hat{R}_j^{(m)}(t+\tau,\mu_1,\mu_2)\hat{D}_j^{(m)}(t)\rangle = \\ &\left(-(\gamma_{{\rm v}j}-i\omega_{{\rm v}j})\mu_1\frac{\partial}{\partial\mu_1}-(\gamma_{{\rm v}j}+i\omega_{{\rm v}j})\mu_2\frac{\partial}{\partial\mu_2}-2\gamma_{{\rm v}j}n_{{\rm v}j}\mu_1\mu_2\right)\langle\hat{R}_j^{(m)}(t+\tau,\mu_1,\mu_2)\hat{D}_j^{(m)}(t)\rangle.\end{aligned} \tag{I.5}$$

with the initial conditions

$$\langle\hat{R}_j^{(m)}(t,\mu_1,\mu_2)\hat{D}_j^{(m)}(t)\rangle = \langle e^{\mu_1\hat{B}_j^{(m)\dagger}(t)}e^{-\mu_2\hat{B}_j^{(m)}(t)}e^{-\Lambda_j(\hat{B}_j^{(m)\dagger}(t)-\hat{B}_j^{(m)})(t)}\rangle \tag{I.6}$$

* vladislavmipt@gmail.com

† anton.zasedatelev@univie.ac.at

One can show that

$$\langle \hat{R}_j^{(m)}(t,\mu_1,\mu_2)\hat{D}_j^{(m)}(t)\rangle = e^{-\Lambda_j^2/2}e^{\mu_2\Lambda_j}e^{-n_{\mathrm{v}j}(\Lambda_j-\mu_1)(\Lambda_j-\mu_2)}. \quad (I.7)$$

From here, we can find the solution to the equation (I.5)

$$\langle \hat{R}_j^{(m)}(t+\tau,\mu_1,\mu_2)\hat{D}_j^{(m)}(t)\rangle = e^{-\Lambda_j^2/2}e^{-n_{\mathrm{v}j}\Lambda_j^2}e^{-n_{\mathrm{v}j}\mu_1\mu_2}e^{n_{\mathrm{v}j}\Lambda_j\mu_1 e^{-(\gamma_{\mathrm{v}j}-i\omega_{\mathrm{v}j})\tau}}e^{(1+n_{\mathrm{v}j})\Lambda_j\mu_2 e^{-(\gamma_{\mathrm{v}j}+i\omega_{\mathrm{v}j})\tau}}. \quad (I.8)$$

Using equality $\langle \hat{D}_j^{(m)\dagger}(t+\tau)\hat{D}_j^{(m)}(t)\rangle = e^{-\Lambda_j^2/2}\langle \hat{R}_j^{(m)}(t+\tau,\Lambda_j,\Lambda_j)\hat{D}_j^{(m)}(t)\rangle$, we finally obtain

$$\langle \hat{D}_j^{(m)\dagger}(t+\tau)\hat{D}_j^{(m)}(t)\rangle = e^{-n_{\mathrm{v}j}\Lambda_j^2}e^{n_{\mathrm{v}j}\Lambda_j^2 e^{-(\gamma_{\mathrm{v}j}-i\omega_{\mathrm{v}j})\tau}}e^{-(1+n_{\mathrm{v}j})\Lambda_j^2}e^{(1+n_{\mathrm{v}j})\Lambda_j^2 e^{-(\gamma_{\mathrm{v}j}+i\omega_{\mathrm{v}j})\tau}}. \quad (I.9)$$

Substitution of Eq. (I.4) and Eq. (I.9) into Eq. (I.3) allows us to transform Eq. (I.1) into

$$I_{\mathrm{em}}(\omega) = \sum_{k'_1,\dots,k'_{N_{\mathrm{vib}}}} z_{k'_1}(n_{\mathrm{v}1}\Lambda_1^2)\dots z_{k'_{N_{\mathrm{vib}}}}(n_{\mathrm{v}N_{\mathrm{vib}}}\Lambda_{N_{\mathrm{vib}}}^2) \sum_{k_1,\dots,k_{N_{\mathrm{vib}}}} z_{k_1}((1+n_{\mathrm{v}1})\Lambda_1^2)\dots z_{k_M}((1+n_{\mathrm{v}N_{\mathrm{vib}}})\Lambda_{N_{\mathrm{vib}}}^2) \frac{1}{N_{\mathrm{mol}}}\sum_{m=1}^{N_{\mathrm{mol}}}\frac{\gamma_{\mathrm{diss}}/2+\gamma_{\mathrm{v}\Sigma}}{(\omega-\omega_0^{(m)}+\omega_{\mathrm{v}\Sigma})^2+(\gamma_{\mathrm{diss}}/2+\gamma_{\mathrm{v}\Sigma})^2}, \quad (I.10)$$

where we introduced

$$\omega_{\mathrm{v}\Sigma} = \sum_{j=1}^{N_{\mathrm{vib}}}\omega_{\mathrm{v}j}(k_j-k'_j), \quad (I.11)$$

$$\gamma_{\mathrm{v}\Sigma} = \sum_{j=1}^{N_{\mathrm{vib}}}\gamma_{\mathrm{v}j}(k_j+k'_j). \quad (I.12)$$

Eq. (I.10) at $T=0$ ($n_{\mathrm{v}j}=0$) coincides with one obtained in [3] via Heisenberg–Langevin approach.

To obtain Eq. (34), we use the arguments from Section 4.1. Namyly, the typical number of illuminated molecules in the organic molecular film is 10^8-10^9. Therefore, the distribution of the energies of the excitons can be treated as continuous. We assume that this distribution is normal with the standard deviation Γ. We also assume that $\Gamma \gg \gamma_{\mathrm{diss}}/2+\gamma_{\mathrm{v}\Sigma}$. In this case, the inhomogeneous broadening leads to the Gauss lineshapes of the spectral peaks [4–8]. Thus, from Eq. (I.10), we obtain Eq. (34).

To obtain Eq. (37) from Eq. (34), we change the sums in Eq. (34) corresponding to the low-frequency vibrations as follows

$$\sum_{n=0}^{+\infty}\frac{e^{-x}x^n}{n!}\dots = \frac{2}{1+\mathrm{efr}\left(\sqrt{x/2}\right)}\frac{1}{\sqrt{2\pi x}}\int_0^{+\infty}dy e^{-(y-x)^2/2x}\dots\Big|_{n\to y} \quad (I.13)$$

II. ABSORPTION SPECTRUM OF AN ORGANIC FILM

The absorption spectrum, $I_{\rm abs}(\omega)$, can be calculated from [9]

$$I_{\rm abs}(\omega_\Omega) = -C_{\rm abs}\sum_{m=1}^{N_{\rm mol}} {\rm Im}\langle\hat{\sigma}^{(m)}(t)e^{i\omega_\Omega t}\rangle/\Omega, \tag{II.1}$$

where $t\to+\infty$ and $C_{\rm abs}$ is the normalization constant. We set $C_{\rm abs}$ such that

$$\int_{-\infty}^{+\infty} I_{\rm abs}(\omega)d\omega = 1. \tag{II.2}$$

In the basis of dressed states, the expression for the absorption spectrum has the form

$$I_{\rm abs}(\omega_\Omega) = -\frac{C_{\rm abs}}{\Omega}\sum_{m=1}^{N_{\rm mol}} {\rm Im}\left\langle \prod_{j=1}^{N_{\rm vib}} \hat{D}_j^{(m)}(t)\hat{S}^{(m)}(t)e^{i\omega_\Omega t}\right\rangle \tag{II.3}$$

The Lindblad equation (31) allows us to write the Heisenberg-Langevin equation for the operator $\hat{S}(t)$

$$\frac{d}{dt}\hat{S}^{(m)}(t) = -(\gamma_{\rm diss}/2 + i\omega_0^{(m)})\hat{S}^{(m)}(t) - i\Omega\hat{D}^{(m)\dagger}(t)e^{-i\omega_\Omega t} + \hat{F}^{(m)}(t), \tag{II.4}$$

where $\hat{F}^{(m)}(t)$ is the noise acting on the dressed electronic state due to the interaction of the molecule with the environment, while $\langle\hat{F}^{(m)}(t)\rangle = 0$. In the equation (II.4), we discarded the term that is nonlinear in $\hat{S}^{(m)}(t)$, assuming that the external field is weak.

We find the operator $\hat{S}(t)$ by integrating the equation (II.4), then we substitute the result into the expression for $I_{\rm abs}(\omega_\Omega)$, and obtain

$$I_{\rm abs}(\omega_\Omega) = C_{\rm abs}\sum_{m=1}^{N_{\rm mol}} {\rm Re}\int_0^{+\infty}\prod_{j=1}^{N_{\rm vib}}\left\langle \hat{D}_j^{(m)}(t)\hat{D}_j^{(m)\dagger}(t')\right\rangle e^{i\omega_\Omega(t-t')}e^{-(\gamma_{\rm diss}/2+i\omega_0)(t-t')}dt'. \tag{II.5}$$

Here, we assume that the noise acting on the dressed electronic state is uncorrelated with the dressed vibrational states of the molecule, i.e. $\langle\hat{D}_j^{(m)}(t)\hat{F}^{(m)}(t)\rangle = \langle\hat{D}_j^{(m)}(t)\rangle\langle\hat{F}^{(m)}(t)\rangle = 0$.

The correlator $\langle\hat{D}_j^{(m)}(t)\hat{D}_j^{(m)\dagger}(t')\rangle$ can be find similarly to how we found $\langle\hat{D}_j^{(m)\dagger}(t+\tau)\hat{D}_j^{(m)}(t)\rangle$ in Section I. Thus, we obtain

$$I_{\rm abs}(\omega) = \sum_{k_1',\dots,k_{N_{\rm vib}}'} z_{k_1'}(n_{\rm v1}\Lambda_1^2)\dots z_{k_{N_{\rm vib}}'}(n_{{\rm v}N_{\rm vib}}\Lambda_{N_{\rm vib}}^2)$$
$$\sum_{k_1,\dots,k_{N_{\rm vib}}} z_{k_1}((1+n_{\rm v1})\Lambda_1^2)\dots z_{k_M}((1+n_{{\rm v}N_{\rm vib}})\Lambda_{N_{\rm vib}}^2)$$
$$\frac{1}{N_{\rm mol}}\sum_{m=1}^{N_{\rm mol}}\frac{\gamma_{\rm diss}/2+\gamma_{\rm v\Sigma}}{(\omega-\omega_0^{(m)}-\omega_{\rm v\Sigma})^2+(\gamma_{\rm diss}/2+\gamma_{\rm v\Sigma})^2}. \tag{II.6}$$

At zero temperature ($n_{vj} = 0$) Eq. (II.6) coincides with the one obtained in [3].

As we did in the Section I we can take into account inhomogeneous broadening and obtain Eq. (35) from Eq. (II.6). Using Eq. (I.13), we can effectively incorporate low-frequency vibrational modes and obtain Eq. (38) from Eq. (35).

III. NUMERICAL SIMULATION OF BOSE–EINSTEIN CONDENSATION

Simulations were carried out within the framework of Lindblad master equation [10]:

$$\frac{d\hat{\rho}}{dt} = \frac{i}{\hbar}\left[\hat{\rho}, \hat{H}\right] + \hat{L}_{up}(\hat{\rho}) + \hat{L}_{low}(\hat{\rho}) + \hat{L}_{vib}(\hat{\rho}) + \hat{L}_{therm}(\hat{\rho}) + \hat{L}_{pump}(\hat{\rho}) + \hat{L}_{seed}(\hat{\rho}) \qquad \text{(III.1)}$$

Tracing out polariton occupation we can adiabatically exclude vibrational degrees of freedom and obtain the following discrete set of rate equations within the region of interest $|k| < 3\mu m^{-1}$.

$$\begin{aligned} \frac{dn_P}{dt} &= -\gamma_P n_P + \kappa_P(t) - \sum_j \Gamma_j^P n_P(n_j + D_j) \\ \frac{dn_i}{dt} &= -\gamma_i n_i + \kappa_{seed}(k_i, t) + \Gamma_i^P n_P(n_i + D_i) + \\ &+ \sum_j \left[\gamma_{therm}^{j\to i} n_j(n_i + D_i) - \gamma_{therm}^{i\to j} n_i(n_j + D_j)\right] \end{aligned} \qquad \text{(III.2)}$$

To access spectral properties we apply quantum regression theorem [10] to generate equations for two-time correlator functions in a form: $\langle \hat{s}^\dagger_{\boldsymbol{k}}(t+\tau)\hat{s}_{\boldsymbol{k}}(t)\rangle$, where amplitudes of polariton states within region of interest defined as $\hat{s}_{\boldsymbol{k}} = \langle \hat{s}_{\boldsymbol{k}}\rangle$ (*mean-field approximation*). On the next step, we solve them numerically and apply Fourier transformation to the two-time correlators at each mode followed by integration over time.

We numerically solve the differential equations for $N = 31$ modes at the lower polariton branch including the ground state. The parameters for the model adopted from experimental data Ref.[11], the main ones are the following:

- Light-matter interaction: $\omega_{exc} = 2.72eV$, $\omega_{cav} = 2.64eV$, $\omega_R = 85meV$, $\alpha_{cav} = 2.2meV * \mu m^2$
- Decay rates: $\gamma_{cav} = 4.4meV$, $\gamma_{exc} = 60meV$
- High-energy vibrations: $\omega_{vib} = 0.199eV$, $\gamma_{vib} = 2.5meV$, $g = 0.5meV$
- Optical pumping: $\omega_p = 2.8eV$, $\tau_P^{FWHM} = 200fs$, $P = 2P_{th}$

- Polariton seed: $k_{seed} = 2.55\mu m^{-1}$, $\sigma_{seed} = 0.2\mu m^{-1}$, $\tau_{seed}^{FWHM} = 200fs$

The estimation Eq. (35) gives the thermalization rate for the neighbouring states with the wave vectors $\mathbf{k}$ and $\mathbf{k}'$

$$\left(\gamma_{\text{therm}}^{\mathbf{k}'\mathbf{k}}\right)_{k_BT \gtrsim \hbar\omega_M}^{\text{est}} \simeq 1.4 \cdot 10^{-8} \text{ (eV)}. \tag{III.3}$$

where we set $A_1 \approx 18$ meV, $A_2 \approx 200$ meV^2 and $\omega_M = 200$ cm^{-1} (see main text Section 5). The numerical simulations ignore some of the details in the thermalization process, accounting for them only effectively. For example, here we assume that thermalization rate is constant within the region of interest, i.e. it does not depend on the in-plane momentum $\hbar k$: $\gamma_{therm}^{\boldsymbol{k}_1 \to \boldsymbol{k}_2} = \gamma_{therm}$ when $\omega_1 > \omega_2$ and is equal to $\gamma_{therm} \exp\left(-\hbar(\omega_2 - \omega_1)/k_BT\right)$ otherwise. Therefore, we cannot use our estimation Eq. (III.3) directly. Nevertheless, by averaging over many thermalization steps of Eq. (III.3), our analytical theory can provide the effective thermalization rate per step of $\gamma_{therm} \simeq 5 \cdot 10^{-10}$ eV that we use in the numerical simulations.

[1] M. O. Scully and S. Zubairy, *Quantum optics* (CambridgeUniversity Press, Cambridge, England, 1997).

[2] H. Carmichael, *An open systems approach to quantum optics: lectures presented at the Université Libre de Bruxelles, October 28 to November 4, 1991*, Vol. 18 (Springer Science & Business Media, 2009).

[3] M. Reitz, C. Sommer, and C. Genes, Langevin approach to quantum optics with molecules, Physical review letters **122**, 203602 (2019).

[4] E. Knapp, Lineshapes of molecular aggregates, exchange narrowing and intersite correlation, Chemical Physics **85**, 73 (1984).

[5] F. C. Spano, J. Clark, C. Silva, and R. H. Friend, Determining exciton coherence from the photoluminescence spectral line shape in poly (3-hexylthiophene) thin films, The Journal of chemical physics **130** (2009).

[6] S. Guha, J. Rice, Y. Yau, C. M. Martin, M. Chandrasekhar, H. R. Chandrasekhar, R. Guentner, P. S. De Freitas, and U. Scherf, Temperature-dependent photoluminescence of organic semiconductors with varying backbone conformation, Physical Review B **67**, 125204 (2003).

[7] R. Borrelli, S. Ellena, and C. Barolo, Theoretical and experimental determination of the absorption and emission spectra of a prototypical indolenine-based squaraine dye, Physical Chemistry Chemical Physics **16**, 2390 (2014).

[8] O. Ostroverkhova, Organic optoelectronic materials: mechanisms and applications, Chemical reviews **116**, 13279 (2016).

[9] R. Loudon, *The quantum theory of light* (OUP Oxford, 2000).

[10] A. V. Zasedatelev, A. V. Baranikov, D. Sannikov, D. Urbonas, F. Scafirimuto, V. Y. Shishkov, E. S. Andrianov, Y. E. Lozovik, U. Scherf, T. Stöferle, *et al.*, Single-photon nonlinearity at room temperature, Nature **597**, 493 (2021).

[11] A. V. Baranikov, A. V. Zasedatelev, D. Urbonas, F. Scafirimuto, U. Scherf, T. Stöferle, R. F. Mahrt, and P. G. Lagoudakis, All-optical cascadable universal logic gate with sub-picosecond operation, arXiv preprint arXiv:2005.04802 https://doi.org/10.48550/arXiv.2005.04802 (2020).
